# F-Value Time-Frequency Analysis: Between-Within Variance Analysis

**DOI:** 10.3389/fnins.2021.729449

**Published:** 2021-12-09

**Authors:** Hong Gi Yeom, Hyundoo Jeong

**Affiliations:** ^1^Department of Electronics Engineering, Chosun University, Gwangju, South Korea; ^2^Department of Mechatronics Engineering, Incheon National University, Incheon, South Korea

**Keywords:** F-value, ANOVA, electroencephalography, analysis of variance, time-frequency analysis

## Abstract

Studies on brain mechanisms enable us to treat various brain diseases and develop diverse technologies for daily life. Therefore, an analysis method of neural signals is critical, as it provides the basis for many brain studies. In many cases, researchers want to understand how neural signals change according to different conditions. However, it is challenging to find distinguishing characteristics, and doing so requires complex statistical analysis. In this study, we propose a novel analysis method, FTF (F-value time-frequency) analysis, that applies the F-value of ANOVA to time-frequency analysis. The proposed method shows the statistical differences among conditions in time and frequency. To evaluate the proposed method, electroencephalography (EEG) signals were analyzed using the proposed FTF method. The EEG signals were measured during imagined movement of the left hand, right hand, foot, and tongue. The analysis revealed the important characteristics which were different among different conditions and similar within the same condition. The FTF analysis method will be useful in various fields, as it allows researchers to analyze how frequency characteristics vary according to different conditions.

## Introduction

Analysis methods for neural signals are essential tools to understand the mechanisms of brain activity (Rubinov and Sporns, 2010; Yeom et al., 2020a). Neural signal analysis has important academic implications and can be used in various fields, such as medicine, psychology, and biomedical engineering. For instance, the brain area that causes seizure activity can be accurately identified by analyzing neural activity patterns (Jeong et al., 2016; Kim et al., 2021). This is critical to ensure that the patient is free from epilepsy following surgery. Moreover, analysis of neural signals can be used to diagnose conditions such as Alzheimer’s disease (Dauwels et al., 2010a,b), attention deficit hyperactivity disorder (ADHD) (Arns et al., 2013; Lenartowicz and Loo, 2014), and schizophrenia (Boutros et al., 2008; Li et al., 2008). Accurate diagnosis of psychological diseases enables prompt and appropriate treatments. Furthermore, movement intention can be predicted by analyzing neural activity (Yeom et al., 2013, 2014, 2020b; Kobler et al., 2020; Mondini et al., 2020; Sosnik and Ben Zur, 2020), and a paralyzed person can control a robot arm according to his or her intentions (Hochberg et al., 2012; Collinger et al., 2013). Thus, patients who cannot move their bodies can control electric devices according to their thought by analyzing neural signals.

However, analysis of neural signals such as electroencephalography (EEG), magnetic encephalography (MEG), electrocorticography (ECoG), and local field potential (LFP) is quite challenging. Neural activity changes over time and has different characteristics depending on frequency band and brain region (Pfurtscheller and Lopes da Silva, 1999). For example, the power of alpha waves (8 – 13 Hz) and beta waves (13 – 30 Hz) decreases in the right motor cortex during movements of the left hand (Blankertz et al., 2008). After such a movement, the power of the beta waves increases (Yeom et al., 2013). Likewise, the power of alpha and beta waves decreases in the left motor cortex during movements of the right hand (Blankertz et al., 2008). Other movements also cause changes in power in related brain areas (Blankertz et al., 2008). A decrease and increase in power are called event-related desynchronization (ERD) and event-related synchronization (ERS), respectively (Pfurtscheller and Lopes da Silva, 1999). Also, importantly, analysis of neural signals is complicated because, even if the neural signals are measured under the same conditions, they are different each time. Therefore, it is difficult to identify which changes are caused by differences between the conditions or other noise effects.

Time-frequency analysis is a powerful method of analyzing the characteristics of neural activity (Roach and Mathalon, 2008; Tzallas et al., 2009; Fraiwan et al., 2012; Herrmann et al., 2014). Time-frequency analysis visualizes the variation in signal power as a color in both the time and frequency domains. Therefore, the method allows intuitive analysis of the characteristics of neural signals in both the time and frequency domains. The method minimizes the effects of noise by repeatedly measuring neural signals and calculating an average of all trials. However, in this process, transitory or non-phase-locked activities can be ignored (Pfurtscheller and Lopes da Silva, 1999). Time-frequency analysis is a primary and general analysis method widely used in neuroscience studies (Wacker and Witte, 2013; Herrmann et al., 2014). However, using time-frequency analysis, it is difficult to identify which characteristics occur according to different conditions. For example, if time-frequency analysis is performed for various conditions, the analyst needs to find the differences while visually comparing the results.

Although there have been many studies to overcome the disadvantages of the time-frequency analysis (Addison et al., 2009; Cohen, 2019), few studies have applied statistical methods to the time-frequency analysis. Some studies applied the analysis of variance (ANOVA) to the wavelet to evaluate the similarity between the simulation model and the actual system (Atkinson et al., 2017, 2018). However, these studies were aimed to evaluate the simulation, and these methods could not provide information on which characteristics differ among different conditions.

Analyzing differences depending on conditions is critical. The analysis can be used to investigate the cause of diverse diseases or diagnose it (Dubreuil-Vall et al., 2020; Sebastian-Romagosa et al., 2020; Lin et al., 2021). Moreover, it is the core technology in the brain-computer interface (BCI), which controls computers or various electronic devices according to various intentions (Gu and Hua, 2021; Panachakel and Ramakrishnan, 2021). If the characteristics of neural signals change a little under the same condition and a lot among different conditions, the characteristics reflect the conditional changes. This is the basic principle underlying the ANOVA (Scheffé, 1999). In this study, we suggest a new analysis method that can easily identify the neural characteristics that reflect conditional changes by applying the F-value of ANOVA to time-frequency analysis. To the best of our knowledge, the proposed method is the first analysis method that presents the statistical difference among conditions in both time and frequency.

## Materials and Methods

### Data Description

This study used publicly available EEG data from the Laboratory of Brain-Computer Interfaces, Graz University of Technology (Tangermann et al., 2012). The data is available at http://www.bbci.de/competition/iv. Details of the data are described in a previous paper (Tangermann et al., 2012). Briefly, EEG measurements were recorded during four imagined movements (left hand, right hand, foot, and tongue). Nine subjects participated in the study, and the subjects were instructed to imagine movements according to visual stimuli on the screen. EEG was measured using 22-channel Ag/AgCl electrodes. Electrooculography (EOG) was measured using 3-channel unipolar electrodes. The left mastoid was used as the reference and the right mastoid was used as the ground. The sampling rate was 250 Hz. The signals were band-pass filtered at 0.5 to 100 Hz. A notch filter at 50 Hz was applied to remove line noise. To evaluate the F-value time-frequency (FTF) analysis method in another case, we also analyzed steady-state visually evoked potential (SSVEP) data. The analysis results of the SSVEP data are described in the Supplementary Material.

### Experimental Paradigm

**FIGURE 1:**
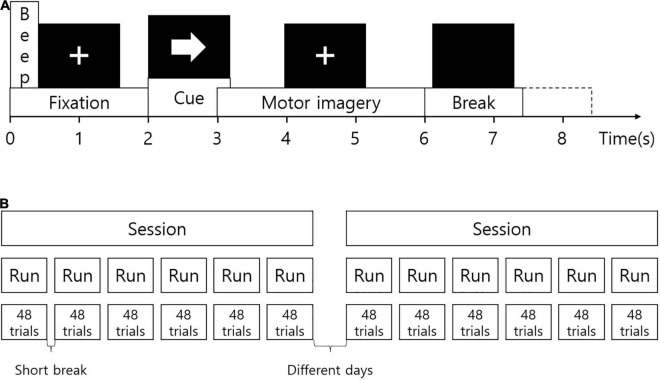
Experimental paradigm. **(A)** The experimental protocol. At the beginning of each trial, a fixation cross was displayed on the screen. A short beep sound was presented together. After 2 s, an arrow pointing in one of the four directions appeared for 1.25 s. The fixation cross disappeared 6 s later. After the fixation cross, a black screen was presented for a short break. **(B)** Configuration of the experiment. EEG signals were measured during two sessions on different days for each subject. One session consisted of 6 runs divided by short breaks. One run included 12 trials for each imagined movement (total, 48 trials).

Subjects were seated in a comfortable chair during the experiment. Visual instruction was presented on a computer screen. At the beginning of each session, EOG signals were measured during eyes open, eyes closed, and eye movements. The recording was approximately 5 min. At the beginning of each trial, a fixation cross was displayed at the center of the screen. A short beep sound was presented together. After 2 s, an arrow pointing in one of the four directions appeared for 1.25 s. The fixation cross disappeared 6 s later. Subjects were instructed to imagine movement according to the direction of the arrow until the cross disappeared. The arrow pointing to the left, right, down, and up corresponded to the imagined movement of the left hand, right hand, foot, and tongue, respectively. After the fixation cross, a black screen was presented for a short break. Figure 1A illustrates the experimental paradigm. EEG signals were measured during two sessions on different days for each subject as shown in Figure 1B. One session is for the training of the prediction model, and the other session is for evaluation. The session for training was used for the analysis to find distinguishing features. One session consisted of 6 runs divided by short breaks. One run included 12 trials for each imagined movement (total, 48 trials). Therefore, each movement was imagined in 72 trials in each session.

### Time-Frequency Analysis

The proposed analysis method is related to time-frequency analysis. To compare the proposed method with time-frequency analysis method, time-frequency analysis was performed. For the time-frequency analysis, EEG signals were epoched. Epoching is segmentation of EEG signals based on an event. The event corresponds to the time at which visual or audible stimuli are given. In this study, EEG signals were epoched from −2 to 4 s relative to presentation of the arrow. Time-frequency power spectra were calculated for each channel of the epoched EEG data using continuous wavelet transform (CWT). To calculate the CWT, complex Morlet wavelet was used. Time-frequency power spectra were normalized by baseline power for each frequency. Baseline corresponded to the recordings taken from −2 to 0 s relative to the arrow cue. Time-frequency power spectra were averaged by trials. All signal processing was performed using MATLAB, 2020b (Mathworks, Natick, MA, United States).

### F-Value Time-Frequency Analysis

**FIGURE 2:**
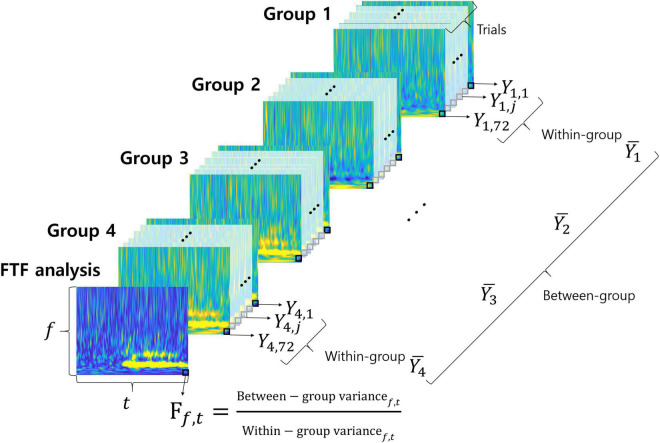
FTF analysis. Each colorful image represents the time-frequency spectrum of a single trial except the front image. The front image shows the results of FTF analysis. Groups 1, 2, 3, and 4 correspond to imagined movements of the left hand, right hand, foot and tongue, respectively. The small black squares are pixels at each time point and frequency.

We suggest a novel F-value time-frequency analysis method. It visualizes the F-value of ANOVA depending on frequency over time, as shown in Figure 2. The F-value is calculated by dividing the variance between groups (among different motor imageries) by the variance within the group (within same imagined movement). A high f-value means a small change within the same condition and a large change among different conditions. In other words, high F-values represent the neural characteristics that vary according to different conditions. F-value time-frequency analysis shows F-values in both the time and frequency domains. Therefore, FTF analysis makes it easy to examine what time and which frequency of the signals are important for classification of the different conditions. The F-value of FTF is calculated as follows:


$$\text{F}=\frac{\text{Between}-\mathrm{group}\text{variance}}{\text{Within}-\mathrm{group}\text{variance}}$$

where between-group variance and within-group variance are calculated as follows:


Between−group variance=∑i=1Kni(Y¯i−Y¯)2K−1

Within−group variance=∑i=1K∑j=1ni(Yij−Y¯i)2N−K

where [$\bar{Y_{i}}$] denotes the mean of samples in the i-th class, [*n_i_*] is the number of samples in the i-th group (type of imagined movement),[$\bar{Y}$] denotes the mean of all 4 groups, [*K*] denotes the number of groups, [*Y*_*ij*_] is the j-th sample in the i-th group and [*N*] is the total number of samples. In this study, the parameters were as follows: [*n*_1_ = *n*_2_ = *n*_3_ = *n*_4_ = 72], [*K* = 4], [*N* = 72*4 = 288]. The F-values are calculated at each time point and frequency, as follows:


$${\text{F}}_{f,t}=\frac{\text{Between}-\text{group}{\text{ variance}}_{f,t}}{\text{Within}-\text{group}{\text{ variance}}_{f,t}}$$

where [*f*] denotes the frequency and [*t*] denotes the time point. In this study, f ranged from 1 to 100 and t ranged from 1 to 1,500 (6 s * 250 sampling rate). F-value for the significance level can be determined by the table of the F-distribution (Beyer, 2019). To find the F-value, a significant level should be determined. On the F-distribution table, the F-value for the significance level is the intersection of degrees of freedom (DOF) between-group and DOF of within-group. Instead of the table, an online calculator is available at www.danielsoper.com/statcalc/calculator.aspx?id = 4.

In this study, DOF of between-group and DOF of within-group were 3 (K-1) and 284 (N-K), respectively. The F-value for the significance level is 3.851286 for probability level 0.01 (*p* = 0.01). Figure 2 illustrates the FTF analysis method. Each of the colorful figures represents the time-frequency spectrum of a single trial except the front one. The front figure shows the results of FTF analysis. Groups 1, 2, 3, and 4 represent the types of imagined movement (left hand, right hand, foot, and tongue, respectively). The small black squares are the pixels at each time point and frequency. MATLAB code of the FTF analysis is available at https://github.com/honggi82/FTF-analysis.

## Results

**FIGURE 3:**
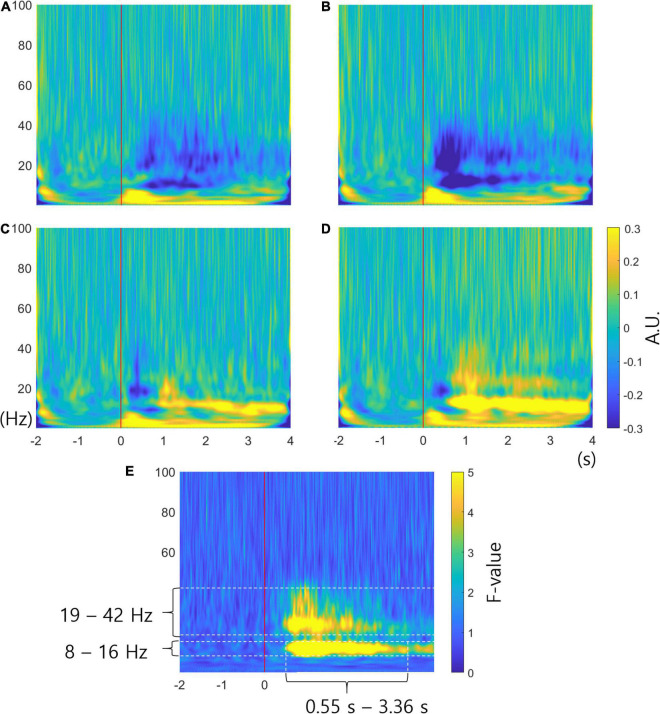
Time-frequency analysis and FTF analysis. **(A–D)** are the averaged time-frequency power spectra for all subjects in channel C3 related to imagined movement of the left hand, right hand, foot and tongue, respectively. The red lines show cue onset. The colors represent the increase or decrease in power in arbitrary units (AUs). **(E)** Averaged FTF analysis for all subjects in channel C3. The unit of FTF analysis is the F-value. The F-value for the significance level is 3.851286 for probability level 0.01 (*p* = 0.01). It is clear that significant differences among conditions occur at 8 – 16 Hz and 19 – 42 Hz. X-axis, time; y-axis, frequency.

Figures 3A–D show the averaged time-frequency power spectra for all subjects in channel C3. Figures 3A–D correspond to the imagined movement of the left hand, right hand, foot, and tongue, respectively. The red lines show the time points at which arrows were presented on the screen. Blue represents a decrease in power (ERD) and yellow represents an increase in power (ERS) compared to baseline. The time-frequency power spectra is given in arbitrary units (AUs) because the spectra were normalized to the baseline. Figure 3A shows ERD at 9 – 40 Hz. Figure 3B shows stronger ERD than Figure 3A at 9 – 40 Hz. Figure 3C reveals a short period of ERD at 15 – 40 Hz and ERS at 10 – 26 Hz after 0.84 s. Figure 3D shows the shortest period of ERD at 15 – 22 Hz, ERS at 9 – 17 Hz after 0.54 s, and weak ERS at 18 – 43 Hz until 3 s. Figures 3A–D generally reveal ERS at 0 – 8 Hz from 0 to about 3.5 s. It is difficult to determine which frequency causes the differences among different conditions. Figure 3E illustrates the FTF analysis in channel C3. The unit of FTF analysis is the F-value. The figure shows significant differences among conditions occur at 8 – 16 Hz and 19 – 42 Hz from 0.55 to 3.36 s. The F-values were low at 0 – 8 Hz, which commonly represented ERS in the time-frequency analysis. The FTF analysis enables examination of which frequency causes the difference. The analysis results mean that ERS of delta and theta waves (0 – 8 Hz) is common among different movements. The analysis also shows that short ERD of beta waves between 0 and 0.5 s is similar among movements. The FTF analysis reveals that ERS or ERD at alpha and beta waves (8 – 42 Hz) are critical features distinguishing different movements.

**FIGURE 4:**
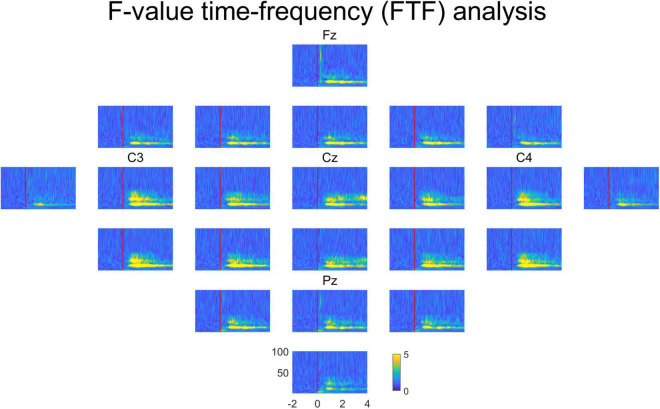
Averaged FTF analysis for all subjects in all channels. Fz, C3, Cz, C4 and Pz are the channel locations in the international 10–20 system. The unit of FTF analysis is the F-value. X-axis, time; y-axis, frequency.

Figure 4 represents the averaged FTF analysis for all subjects in all channels. The multi-channel FTF analysis enables researchers to examine the overall characteristics at one time easily. Fz, C3, Cz, C4, and Pz are the channel locations in the international 10–20 system. Channels C3, Cz, and C4 show high F-values. Channel Cz is close to the brain area responsible for foot movement. The brain area responsible for right-hand movement is close to channel C3, and the brain area responsible for left-hand movement is close to channel C4. Therefore, FTF analysis reveals the areas that play a distinct role among different movements.

**FIGURE 5:**
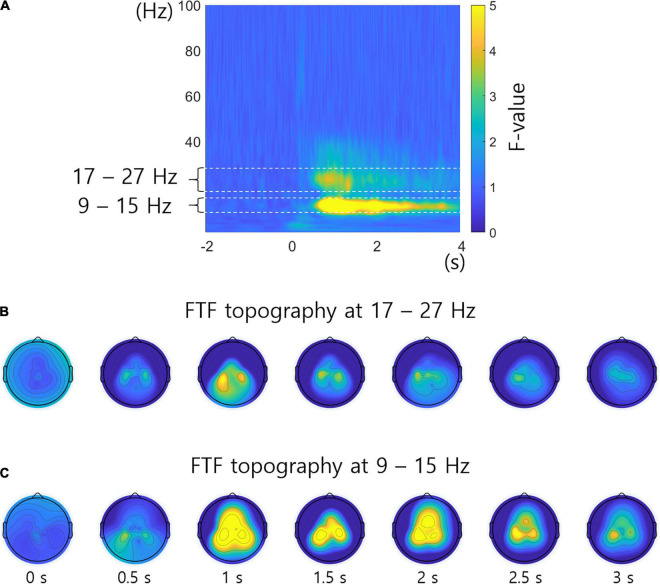
F-value topography in specific frequency bands. **(A)** FTF analysis averaged across all subjects and channels. The 17 – 27 Hz and 9 – 15 Hz frequency bands show significant F-values. **(B)** F-value topography over time at 17 – 27 Hz. **(C)** F-value topography over time at 9 – 15 Hz. The unit of topography is the F-value.

Figure 5A shows the FTF analysis averaged across all subjects and channels. Figures 5B,C represent the F-values of the FTF analysis by topography over time at 17 – 27 Hz and 9 – 15 Hz, which have significant F-values in Figure 5A. Crucial neural characteristics that differ among the different conditions in frequency, time, or location are readily examined by the FTF analysis. Figures 5B,C reveal high F-values in the areas related to the hands and foot.

## Discussion

### F-Value Time-Frequency-Analysis Visualizes Statistical Difference of Neural Signals Among Different Tasks

Although time-frequency analysis is a powerful method of analyzing the characteristics of neural activity, it is difficult to identify which characteristics occur according to the different conditions. On the other hand, the proposed FTF analysis method represents the neural characteristics that vary according to different conditions using statistical values. As shown in Figures 3A–D, there are common changes and different changes across conditions. Therefore, it is difficult to examine the difference among tasks intuitively by time-frequency analysis. FTF-analysis showed high F-values when neural activities were different across conditions and low F-values when the neural activities were similar in Figure 3E. F-values at 8 – 16 Hz and 19 – 42 Hz were significantly high. The frequencies 8 – 16 Hz and 19 – 42 Hz approximately correspond to alpha and beta waves, respectively. It is well-known that movements of different body parts produce different ERD and ERS patterns at alpha and beta waves (Blankertz et al., 2007, 2008; Tangermann et al., 2012). Figures 3A–D represent different ERD and ERS at 8 – 16 Hz and 19 – 42 Hz. Figures 3A,B shows weak and strong ERD, respectively. Figures 3C,D reveals weak and strong ERS, respectively. Therefore, there are differences across conditions at 8 – 16 Hz and 19 – 42 Hz. On the other hand, F-values at 0 – 8 Hz were low because ERS was common in Figures 3A–D. Moreover, ERD was commonly observed at 15 – 20 Hz at 0 – 0.5 s. Therefore, the F-values were low at 15 – 20 Hz at 0 – 0.5 s.

Although F-values were high at broad areas in Figure 4 because of volume conduction, F-values were higher at channels C3, C4, and Cz than other channels. In the case of EEG, it is difficult to say that the EEG signal at a specific location represents the response of a specific brain area. However, the C3, C4, and Cz channels are close to the brain areas responsible for the right hand, left hand, and foot movements, respectively (Seeck et al., 2017). Moreover, neural responses to the right hand, left hand, and foot movements, are often observed at C3, C4, Cz (Blankertz et al., 2007, 2008; Tangermann et al., 2012). Therefore, it means that FTF analysis shows well the neural characteristics that vary according to different conditions in time, frequency, and channel. The neural characteristics of imagined tongue movements were not clearly observed with time-frequency analysis and FTF analysis. It seems that the tongue-related area of the brain may be more lateral than the measured channels (de Klerk et al., 2015; Wennberg et al., 2019).

Although we analyzed one case of data, the results showed that the proposed FTF analysis effectively represents the differences of neural signals in time, frequency, or area among conditions. We plan to apply FTF analysis to neural signals in various cases through future studies.

### Time-Frequency Analysis and F-Value Time-Frequency Analysis Are Complementary

Although FTF analysis identifies neural characteristics that change according to different conditions, it does not mean that the FTF analysis is better than the time-frequency analysis. Time-frequency analysis is especially useful for analyzing neural activity under one condition. Figures 3A–D show different ERD and ERS patterns depending on the type of imagined movement. Therefore, time-frequency analysis provides valuable information on a specific condition, such as imagined movement of the left hand, whereas FTF is a powerful tool for finding characteristics that differ depending on multiple conditions. Therefore, time-frequency analysis and FTF analysis can be used complementary to each other. FTF analysis can be used in various studies, including those related to the brain but also those related to sound, communication, and so on (Akansu et al., 2010; Varanis et al., 2021).

### F-Value Time-Frequency Analysis Has the Disadvantages of Time-Frequency Analysis

F-Value Time-Frequency (FTF) analysis uses time-frequency analysis for F-value calculation. Any time-frequency analysis can be used for the FTF analysis because the F-values are calculated among values of time-frequency analysis. Not only traditional methods but also recently proposed methods also can be applied to the FTF analysis (Cohen, 2019; Yang et al., 2019; Liu et al., 2020; Varanis et al., 2021). However, FTF analysis has the disadvantages of time-frequency analysis because it is based on time-frequency analysis. Time-frequency analysis requires a trade-off between time resolution and frequency resolution (Grochenig, 2001). It means that based on the uncertainty principle, the frequency resolution decreases to increase the time resolution, and the time resolution decreases to increase the frequency resolution. Short-time Fourier transform (STFT) uses fixed resolution in time and frequency (Varanis et al., 2021). However, high-frequency requires high-time resolution and low-frequency requires low-time resolution. CWT increases temporal resolution as frequency increases using a wavelet (Varanis et al., 2021). Time-frequency analysis can be obtained by calculating the absolute values of the CWT. Generally, the CWT’s absolute values are calculated for each trial’s signals and then averaged by trials. The process for calculating the absolute values removes the phasor information. Therefore, even if there is important information in the phase, it is difficult to know with time-frequency analysis. Furthermore, the averaging process reduces the transitory or non-phase locked activities, although the process diminishes the noise.

## Conclusion

In this study, we suggest a novel analysis method that can be used to easily identify neural characteristics that reflect conditional changes by applying the F-value of ANOVA to time-frequency analysis. F-value time-frequency analysis represents the statistical differences among conditions in both the time and frequency domains. EEG signals during 4 movement imagination tasks were analyzed by the FTF method. It was easy to observe critical characteristics that differed in terms of time, frequency, and location. The FTF method will be useful in various fields that analyze how frequency characteristics vary according to different conditions.

## Data Availability Statement

Publicly available datasets were analyzed in this study. This data can be found here: http://www.bbci.de/competition/iv.

## Ethics Statement

Ethical review and approval were not required for the study on humans in accordance with the local legislation and institutional requirements. Written informed consent for participation was not required for this study in accordance with the national legislation and the institutional requirements.

## Author Contributions

HY proposed and programmed the FTF algorithm. HY and HJ wrote and reviewed the manuscript. Both authors contributed to the article and approved the submitted version.

## Conflict of Interest

The authors declare that the research was conducted in the absence of any commercial or financial relationships that could be construed as a potential conflict of interest.

## Publisher’s Note

All claims expressed in this article are solely those of the authors and do not necessarily represent those of their affiliated organizations, or those of the publisher, the editors and the reviewers. Any product that may be evaluated in this article, or claim that may be made by its manufacturer, is not guaranteed or endorsed by the publisher.
