# Supplementary material for: F-Value Time-Frequency Analysis: Between-Within Variance Analysis
Source: Front Neurosci. 2021 Dec 9;15:729449. doi: 10.3389/fnins.2021.729449 (PMC8697975; doi:10.3389/fnins.2021.729449)
Supplement: Supplementary file 1 [file Image_1.PDF]

Supplementary Material

Data

To evaluate the FTF analysis method in another case, we analyzed steady-state visually evoked potential (SSVEP) data. The EEG data is publicly available at http://dx.doi.org/10.5524/100542. Details of the data are described in a previous paper (Lee et al., 2019). Briefly, fifty-four healthy subjects (ages 24-35; 25 females) participated in the experiment. The subjects were seated in front of an LCD monitor. Four SSVEP stimuli flickering at 5.45, 6.67, 8.57, and 12 Hz were presented on a monitor. Each stimulus was shown at down, right, left, and up, respectively. At the beginning of each trial, participants were instructed to see the center of a screen. The target stimulus was presented in yellow, and other stimuli were shown in white on the black screen for 4 s. After the target presentation, subjects were asked to see the flickering target stimulus for 4s. After the stimulus, a black screen was presented for 2 s. This sequence was repeated 25 times. Therefore, 100 trials (4 classes × 25 trials) were recorded for a training session. Another 100 trials for the test were measured in the real-time experiment. Therefore, 200 trials for each subject were used. EEG signals were recorded using the 62 channel EEG system with a sampling rate of 1,000 Hz.

Analysis

The SSVEP data was analyzed using time-frequency analysis and FTF analysis as described in the manuscript. In this case, DOF of between-group and DOF of within-group were 3 (K-1) and 196 (N-K), respectively. The F-value for the significance level is 3.883084 for probability level 0.01 ($p = 0.01$).

Results

Supplementary Fig. 1 shows the Time-frequency analysis and FTF analysis of the SSVEP data. Supplementary Fig. 1 (A) – (D) are the averaged time-frequency power spectra for all subjects in

channel Oz. Supplementary Fig. 1 (A) – (D) correspond to the stimuli flickering at 5.45, 6.67, 8.57, and 12 Hz, respectively. Supplementary Fig. 1 (E) is the averaged FTF analysis for all subjects in channel Oz. Supplementary Fig. 1 (A) – (D) represent high power at 5.45, 6.67, 8.57, and 12 Hz, and its harmonic frequency, respectively. Supplementary Fig. 1 (E) shows high F values between 4-30 Hz, including 5.45-12 Hz and harmonic frequency. Although FTF analysis could not show characteristics of each case, it represents characteristics that reflect conditional changes. Supplementary Fig. 2 is averaged FTF analysis for all subjects in all channels. The PO3, POz, PO4, O1, Oz, and O2 channels show significantly high F values. These channels are close to the visual cortex.

Supplementary References

Lee, M.H., Kwon, O.Y., Kim, Y.J., Kim, H.K., Lee, Y.E., Williamson, J., Fazli, S., and Lee, S.W. (2019). EEG dataset and OpenBMI toolbox for three BCI paradigms: an investigation into BCI illiteracy. *Gigascience* 8.

Supplementary Figures

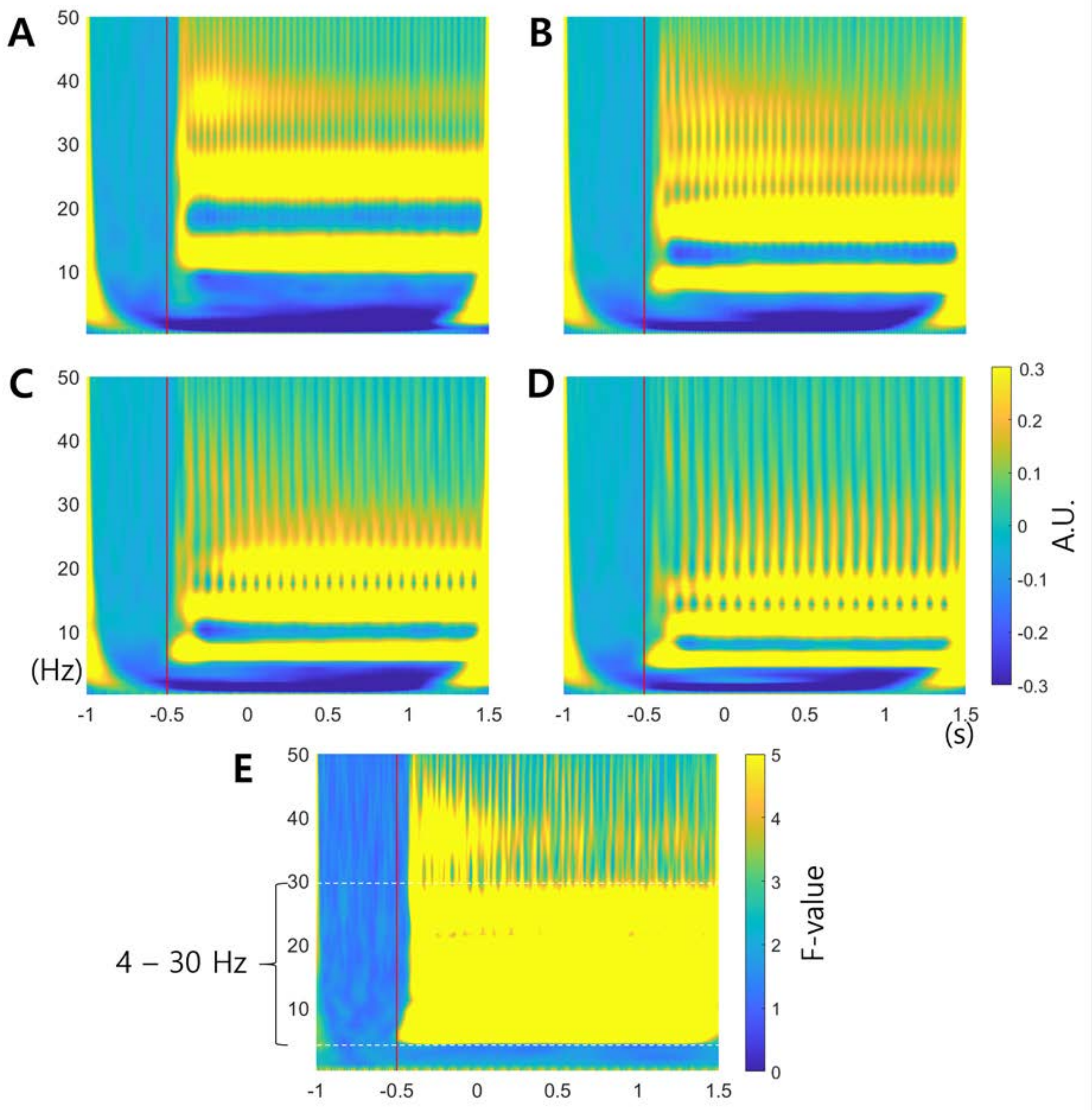

Supplementary Figure 1: Time-frequency analysis and FTF analysis. (A) – (D) are the averaged time-frequency power spectra for all subjects in channel Oz. Fig. 1 (A) – (D) correspond to the stimuli flickering at 5.45, 6.67, 8.57, and 12 Hz, respectively. The red lines show cue onset. The colors represent the increase or decrease in power in arbitrary units (AUs). (E) Averaged FTF analysis for all subjects in channel Oz. It shows high F values between 4-30 Hz, including 5.45-12 Hz and harmonic frequency.

The unit of FTF analysis is the F-value. The F-value for the significance level is 3.851286 for probability level 0.01 (p = 0.01).

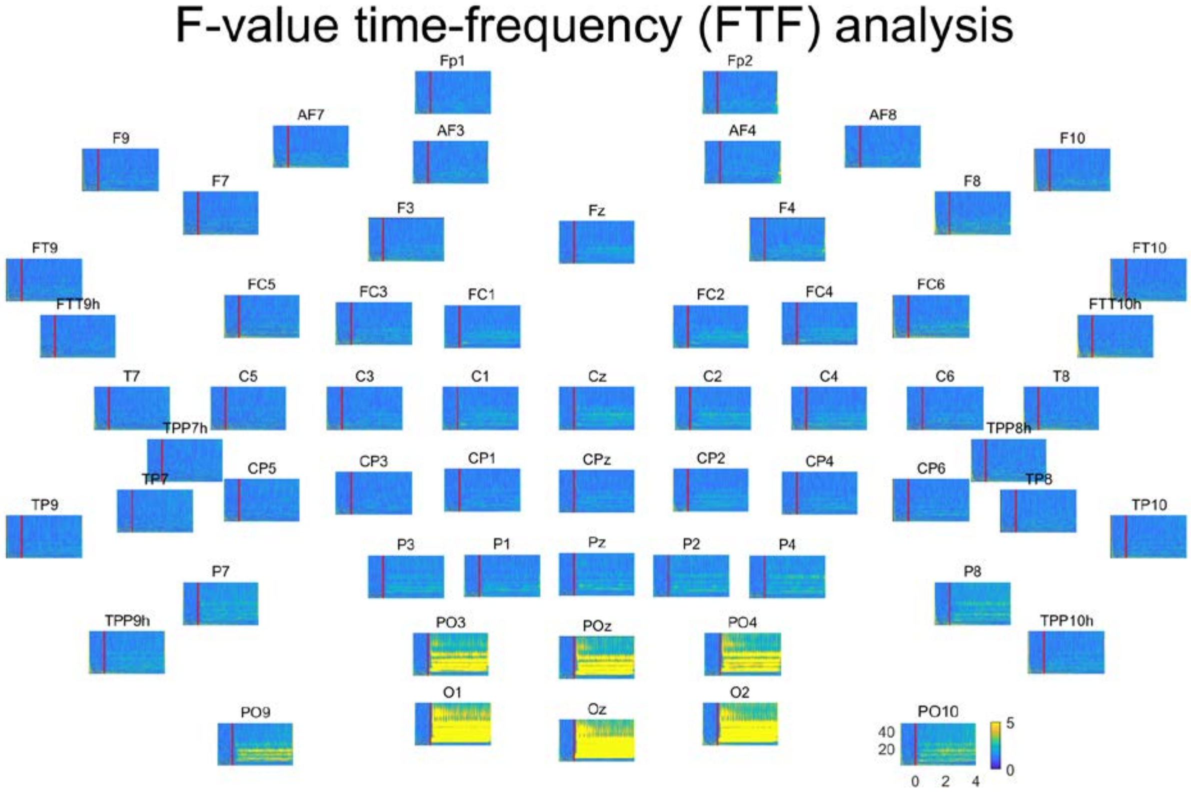

Supplementary Figure 2: Averaged FTF analysis for all subjects in all channels. The unit of FTF analysis is the F-value. X-axis, time; y-axis, frequency.
